# Biodegradable and Stimuli-Responsive Nanomaterials for Targeted Drug Delivery in Autoimmune Diseases

**DOI:** 10.3390/jfb16010024

**Published:** 2025-01-14

**Authors:** Nargish Parvin, Sang Woo Joo, Tapas K. Mandal

**Affiliations:** School of Mechanical Engineering, School of Basic Science, Yeungnam University, Gyeongsan 38541, Republic of Korea; nargish.parvin@gmail.com

**Keywords:** biodegradable nanomaterials, stimuli-responsive drug delivery, autoimmune disease treatment, targeted drug delivery systems, immunomodulation

## Abstract

Autoimmune diseases present complex therapeutic challenges due to their chronic nature, systemic impact, and requirement for precise immunomodulation to avoid adverse side effects. Recent advancements in biodegradable and stimuli-responsive nanomaterials have opened new avenues for targeted drug delivery systems capable of addressing these challenges. This review provides a comprehensive analysis of state-of-the-art biodegradable nanocarriers such as polymeric nanoparticles, liposomes, and hydrogels engineered for targeted delivery in autoimmune therapies. These nanomaterials are designed to degrade safely in the body while releasing therapeutic agents in response to specific stimuli, including pH, temperature, redox conditions, and enzymatic activity. By achieving localized and controlled release of anti-inflammatory and immunosuppressive agents, these systems minimize systemic toxicity and enhance therapeutic efficacy. We discuss the underlying mechanisms of stimuli-responsive nanomaterials, recent applications in treating diseases such as rheumatoid arthritis, multiple sclerosis, and inflammatory bowel disease, and the design considerations essential for clinical translation. Additionally, we address current challenges, including biocompatibility, scalability, and regulatory hurdles, as well as future directions for integrating advanced nanotechnology with personalized medicine in autoimmune treatment. This review highlights the transformative potential of biodegradable and stimuli-responsive nanomaterials, presenting them as a promising strategy to advance precision medicine and improve patient outcomes in autoimmune disease management.

## 1. Introduction

Autoimmune diseases represent a complex group of disorders in which the immune system mistakenly attacks the body’s own healthy tissues, leading to chronic inflammation and long-term damage to vital organs [1]. These diseases, which include conditions such as rheumatoid arthritis (RA), multiple sclerosis (MS), lupus, and inflammatory bowel disease (IBD), affect millions of individuals worldwide [2]. The pathogenesis of autoimmune diseases is multifactorial, involving genetic predispositions, environmental factors, and dysregulation of immune responses [3]. While these diseases share a common feature of immune system dysfunction, their clinical presentations and mechanisms vary significantly, making treatment particularly challenging [4]. The immune system in healthy individuals can differentiate between self and non-self, ensuring tolerance to the body’s own cells and tissues. However, in autoimmune diseases, this tolerance is lost, leading to an overactive immune response that damages normal tissues. For example, in RA, the immune system attacks the synovial joints, resulting in painful inflammation, cartilage degradation, and eventual joint destruction [5]. In MS, the immune system targets the central nervous system, causing demyelination and disruption of nerve function. In IBD, there is a chronic inflammatory response in the gastrointestinal tract, leading to pain, diarrhea, and possible life-threatening complications [6]. Effective management of autoimmune diseases remains a significant challenge, as the therapeutic options currently available are limited in terms of specificity and effectiveness. Most traditional therapies rely on broad immunosuppressive agents, including corticosteroids and disease-modifying antirheumatic drugs (DMARDs), which are designed to dampen the immune response and reduce inflammation. Although these treatments can provide symptomatic relief, they are often associated with serious side effects, including increased susceptibility to infections, cardiovascular issues, gastrointestinal damage, and bone loss [7]. Moreover, these treatments generally lack specificity, leading to systemic immunosuppression that compromises the immune system’s ability to combat infections and other diseases. The need for therapies that can specifically target the affected tissues while minimizing systemic effects is critical in improving the clinical outcomes of patients with autoimmune diseases. Recent advances in nanotechnology have opened new avenues for the treatment of autoimmune diseases. Nanotechnology involves the use of materials at the nanoscale (typically 1–100 nm) to design novel drug delivery systems that can offer improved targeting, stability, and controlled release of therapeutic agents [8].

The unique properties of nanomaterials—such as their small size, large surface area, and ability to be functionalized with various targeting ligands—make them ideal candidates for addressing the challenges of autoimmune disease treatment. Nanoparticles, liposomes, and hydrogels are some of the most widely explored nanomaterials in drug delivery. These nanocarriers can be designed to encapsulate therapeutic agents, such as anti-inflammatory drugs, cytokines, or biologics, and deliver them to the site of inflammation, reducing the need for systemic distribution and limiting side effects. In particular, biodegradable and stimuli-responsive nanomaterials have garnered considerable attention in recent years [9]. Biodegradable nanomaterials can safely degrade within the body into non-toxic byproducts, which eliminates concerns about long-term accumulation in tissues and organs. Stimuli-responsive systems, on the other hand, can release their payloads in response to specific environmental cues, such as changes in pH, temperature, redox potential, or enzymatic activity, which are commonly altered in the inflamed tissues of autoimmune disease sites [10,11,12]. This ability to control the release of therapeutic agents in a spatially and temporally controlled manner enhances the effectiveness of treatments and minimizes off-target effects. By combining biodegradability with stimuli-responsiveness, these nanomaterials offer a promising strategy to improve the precision and efficacy of drug delivery in autoimmune diseases [13,14]. In this review, we explore the current advancements in biodegradable and stimuli-responsive nanomaterials for targeted drug delivery in the treatment of autoimmune diseases. We discuss the design, mechanisms, and potential applications of these nanomaterials in autoimmune therapies, focusing on diseases such as rheumatoid arthritis, multiple sclerosis, and inflammatory bowel disease. Furthermore, we examine the challenges that remain in the clinical translation of these technologies, including issues related to biocompatibility, scalability, and regulatory approval, and provide insights into the future directions for integrating advanced nanomaterials into personalized medicine for autoimmune disease management.

The immune response in RA is primarily mediated by T cells and macrophages, which produce pro-inflammatory cytokines such as tumor necrosis factor (TNF) and interleukin-1 (IL-1), contributing to tissue damage and joint degradation [15]. Multiple sclerosis (MS) is another prominent autoimmune disease, but it primarily affects the central nervous system (CNS). In MS, the immune system targets the myelin sheath, which is the protective covering of nerve fibers in the brain and spinal cord. This immune attack leads to demyelination, impaired nerve conduction, and the formation of scar tissue (sclerosis), causing neurological symptoms such as weakness, numbness, vision problems, and cognitive decline. The pathogenesis of MS involves both genetic and environmental factors, with the immune system’s activation of autoreactive T cells playing a central role in the disease. The exact cause of MS remains unclear, but viral infections, particularly Epstein–Barr virus (EBV), have been implicated as potential triggers for disease onset [16]. Inflammatory bowel disease (IBD) encompasses two primary conditions—Crohn’s disease and ulcerative colitis—both of which involve chronic inflammation of the gastrointestinal tract. In Crohn’s disease, inflammation can occur anywhere along the digestive tract, from the mouth to the anus, whereas ulcerative colitis is confined to the colon and rectum. The cause of IBD is thought to involve an abnormal immune response to the gut microbiota in genetically predisposed individuals. This immune dysregulation leads to the infiltration of immune cells into the intestinal mucosa, causing chronic inflammation, ulceration, and, in severe cases, bowel perforation or cancer. IBD symptoms include abdominal pain, diarrhea, weight loss, and fatigue, which can significantly impact the quality of life of affected individuals [17]. Despite advances in the understanding of autoimmune diseases, effective treatments remain limited. The use of non-specific immunosuppressive therapies has been the dominant approach, but these therapies are often associated with systemic side effects that can worsen patients’ conditions in the long term. Novel approaches involving targeted drug delivery, particularly those utilizing biodegradable and stimuli-responsive nanomaterials, are gaining prominence as they promise to overcome these challenges by delivering drugs directly to the site of disease while minimizing harmful systemic effects.

### 1.1. Current Challenges in Autoimmune Disease Treatment

The treatment of autoimmune diseases is fraught with numerous challenges due to the complex and diverse nature of these disorders. While the current therapeutic strategies provide symptomatic relief, they often fail to address the underlying mechanisms of immune dysregulation and inflammation. The majority of conventional treatments focus on suppressing the immune system as a whole, but these broad immunosuppressive approaches come with significant drawbacks. This section outlines the key challenges in autoimmune disease treatment, highlighting the limitations of current therapies and the need for more targeted and effective strategies.

#### 1.1.1. Lack of Specificity in Current Treatments

One of the major challenges in the treatment of autoimmune diseases is the lack of specificity in most conventional therapies. Traditional drugs, such as corticosteroids and nonsteroidal anti-inflammatory drugs (NSAIDs), are designed to reduce inflammation and immune activation systemically. However, these treatments do not discriminate between the immune response against the body’s own tissues and the immune defense against pathogens. As a result, they suppress the immune system indiscriminately, which can leave patients more susceptible to infections, malignancies, and other diseases. Furthermore, such systemic immunosuppression can exacerbate other autoimmune conditions, leading to a cascade of adverse effects that compromise patient health over time [18]. In autoimmune diseases like rheumatoid arthritis and systemic lupus erythematosus (SLE), the immune system’s aberrant activation and inability to differentiate self from non-self leads to persistent inflammation and tissue damage. While biologics that target specific immune pathways (e.g., TNF inhibitors or IL-6 inhibitors) have emerged, they still cannot fully resolve the disease or prevent relapses, and they may not be effective for all patients. Moreover, these therapies often require parenteral administration (e.g., injections or infusions), which can be inconvenient and costly for patients.

#### 1.1.2. Chronicity and Variability of Autoimmune Diseases

Autoimmune diseases are chronic in nature, often requiring long-term management to control symptoms and prevent disease progression. The variable disease course—characterized by periods of exacerbation (flare-ups) and remission—further complicates treatment. During flare-ups, the inflammatory process can lead to severe tissue damage and organ dysfunction, which may be irreversible if not controlled promptly. However, long-term use of potent immunosuppressive drugs can cause side effects, including osteoporosis, metabolic disturbances, and cardiovascular problems. Balancing the need for effective inflammation control while minimizing the risk of long-term side effects is a delicate challenge in autoimmune disease management [19]. Moreover, autoimmune diseases often present with diverse clinical manifestations, which vary not only between diseases but also between patients with the same diagnosis. For example, two patients with rheumatoid arthritis may experience different symptoms, including varying degrees of joint involvement and extra-articular manifestations such as lung and heart issues. This variability complicates the development of standardized treatment regimens and calls for more personalized approaches in managing autoimmune diseases.

#### 1.1.3. Adverse Effects of Long-Term Immunosuppressive Therapy

Long-term use of immunosuppressive drugs, such as methotrexate, corticosteroids, and biologics, can lead to a range of adverse effects, making them less than ideal for long-term treatment. These side effects include increased susceptibility to infections, which is a significant concern in immunocompromised patients, as they are more vulnerable to opportunistic infections like tuberculosis and pneumonia. In addition, the use of corticosteroids can result in weight gain, high blood pressure, diabetes, and osteoporosis, making them unsuitable for long-term use, especially in young or elderly patients [20]. Biologic agents, though effective in some cases, are expensive and require parenteral administration, which can be inconvenient for patients who need frequent treatment. The emergence of drug resistance, as seen with the long-term use of TNF inhibitors in certain autoimmune diseases, further limits the efficacy of these therapies and underscores the need for novel treatment modalities.

#### 1.1.4. Need for Personalized Medicine

Given the complex pathophysiology and the highly variable responses to treatment among autoimmune disease patients, there is an urgent need for personalized or precision medicine. Personalized medicine involves tailoring treatments based on an individual’s genetic makeup, disease phenotype, and response to previous therapies. The aim is to identify specific biomarkers or disease signatures that can predict therapeutic efficacy and minimize adverse effects. Advances in genomics, proteomics, and immunology are paving the way for more personalized treatment approaches that could revolutionize the management of autoimmune diseases. However, translating these advances into clinically viable solutions remains challenging due to the complexity of autoimmune diseases and the need for robust clinical validation of biomarkers and treatment strategies [21].

#### 1.1.5. Clinical Translation and Accessibility

**Figure 1:**
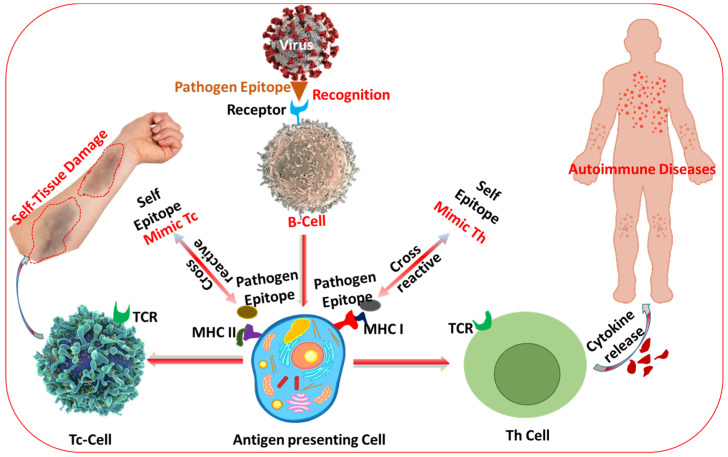
The role of molecular mimicry in infection-induced autoimmunity. Legend: MHC (Major Histocompatibility Complex); TCR (T Cell Receptor).

Another significant challenge in autoimmune disease treatment is the clinical translation of innovative therapies, including those based on nanotechnology. While promising preclinical data often demonstrate the potential for targeted drug delivery systems to enhance the specificity and efficacy of treatments, translating these findings into clinically approved therapies is a lengthy and expensive process. Regulatory hurdles, manufacturing scalability, and concerns regarding biocompatibility and toxicity complicate the successful transition from laboratory to clinical settings. Ensuring that new therapies are affordable, accessible, and safe for a broad population of patients is critical for their widespread adoption. So the current treatment options for autoimmune diseases face numerous challenges, including lack of specificity, chronicity, adverse effects, and variability between patients. As a result, there is an increasing need for more targeted and personalized therapeutic strategies that can overcome these limitations and provide better outcomes for patients with autoimmune diseases. Figure 1 illustrates the process of infection-induced autoimmunity driven by molecular mimicry [22]. In this mechanism, foreign antigens from pathogens share structural similarities with host proteins, leading to immune cross-reactivity. When antigen-presenting cells present pathogen-derived peptides on major histocompatibility complex (MHC) molecules, T cell receptors (TCRs) recognize these peptides and initiate an immune response. However, due to the molecular resemblance between pathogen antigens and self-proteins, the activated immune cells may mistakenly target healthy tissues, resulting in autoimmune disorders. This phenomenon highlights the delicate balance of immune tolerance and the potential for infections to trigger autoimmune responses through shared molecular patterns. The visualization emphasizes the critical roles of MHC and TCR interactions in this pathological process, providing insight into how infections may contribute to the onset of autoimmune diseases.

### 1.2. Overview of Nanotechnology in Drug Delivery

Nanotechnology is an interdisciplinary field that involves the design, fabrication, and application of materials at the nanoscale (typically ranging from 1 to 100 nm). Nanomaterials have unique properties—such as high surface area, small size, and enhanced reactivity—that enable them to interact with biological systems in ways that conventional materials cannot. These properties make nanomaterials particularly useful in the development of advanced drug delivery systems, which are capable of overcoming many of the limitations of traditional therapies. This section provides an overview of the role of nanotechnology in drug delivery, with a specific focus on its potential applications in the treatment of autoimmune diseases.

#### 1.2.1. Principles of Nanotechnology in Drug Delivery

Nanoparticles are defined as particles with at least one dimension in the size range of 1 to 100 nanometers. Nanotechnology-based drug delivery systems are designed to improve the pharmacokinetics, bioavailability, and therapeutic efficacy of drugs. The key advantage of using nanomaterials as drug carriers is their ability to encapsulate therapeutic agents, protect them from degradation, and deliver them in a controlled manner to specific target sites [23]. Nanocarriers can be made from various materials, including lipids, polymers, and metals, and can take the form of nanoparticles, micelles, liposomes, dendrimers, and hydrogels. The ability to modify the surface properties of nanoparticles allows for the attachment of targeting ligands, such as antibodies, peptides, or aptamers, which can direct the nanocarriers to specific tissues or cells [24]. This targeted delivery minimizes the off-target effects often seen with traditional therapies, which are distributed systemically. Additionally, nanoparticles can be designed to release their cargo in response to specific stimuli, such as changes in pH, temperature, or the presence of specific enzymes, providing a level of control over the release of drugs at the site of disease [25].

#### 1.2.2. Types of Nanomaterials Used in Drug Delivery

Several types of nanomaterials have been developed for drug delivery applications. The most commonly used nanomaterials include:

➢ Polymeric Nanoparticles: These are made from biocompatible and biodegradable polymers such as poly(lactic-co-glycolic acid) (PLGA) and poly(lactic acid) (PLA). These nanoparticles are capable of encapsulating a wide range of therapeutic agents, including small molecules, proteins, and nucleic acids. They can be engineered to release drugs in a sustained or controlled manner, thereby reducing the need for frequent dosing and improving therapeutic outcomes [26].
➢ Liposomes: Liposomes are spherical vesicles made of lipid bilayers, which can encapsulate both hydrophobic and hydrophilic drugs. Liposomes are widely used for the delivery of chemotherapy agents, but they also hold promise in autoimmune diseases due to their ability to enhance drug stability, target specific tissues, and reduce side effects [27].

#### 1.2.3. Biodegradable and Stimuli-Responsive Nanomaterials

**Figure 2:**
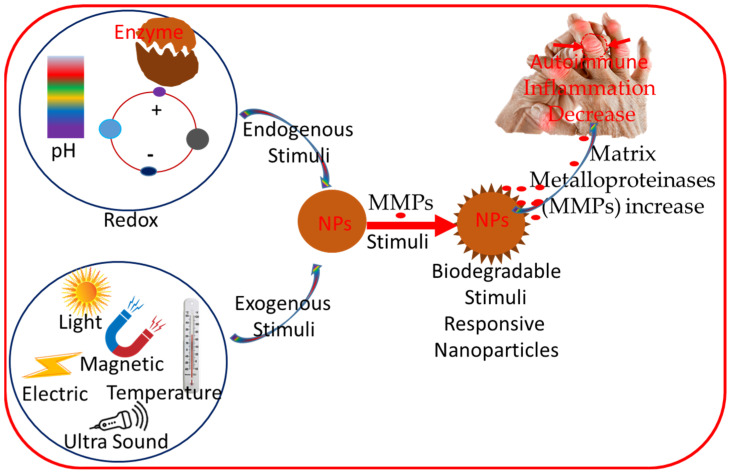
Various stimuli-responsive agents and mechanisms of stimuli-responsive nanomaterials for controlled drug release to manage autoimmune inflammation.

Biodegradable and stimuli-responsive nanomaterials have gained significant attention in recent years due to their ability to improve the targeting and controlled release of therapeutic agents. Biodegradable nanomaterials are designed to degrade within the body into non-toxic byproducts, which reduces concerns regarding the accumulation of nanoparticles in organs and tissues. This is particularly important in the context of autoimmune diseases, where long-term treatment is often necessary. Stimuli-responsive nanomaterials can release their payload in response to specific triggers in the body, such as changes in pH, temperature, or the presence of certain enzymes. For example, in the case of autoimmune diseases, the inflammatory microenvironment often exhibits altered pH or increased levels of specific enzymes, such as matrix metalloproteinases (MMPs) (Figure 2). Nanomaterials that can respond to these stimuli enable localized drug release at the site of inflammation, thereby enhancing therapeutic efficacy while minimizing systemic toxicity [28].

#### 1.2.4. Applications of Nanotechnology in Autoimmune Disease Treatment

**Figure 3:**
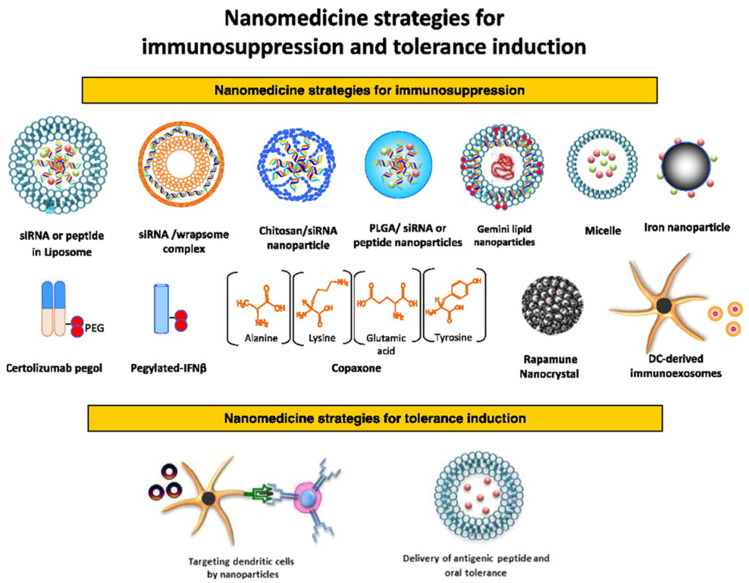
The use of nanomedicine in treating autoimmune diseases: advancing from immunosuppression to promoting immune tolerance. Adapted with permission from Ref. [32]. Copyright 2015 Elsevier.

Nanotechnology has shown significant promise in the treatment of autoimmune diseases. For instance, in rheumatoid arthritis, nanoparticles have been used to deliver anti-inflammatory drugs or biologics directly to the inflamed joints, reducing the need for systemic immunosuppressive therapy and improving drug efficacy [29]. Similarly, in multiple sclerosis, nanocarriers have been designed to cross the blood–brain barrier (BBB) and deliver immunomodulatory agents to the central nervous system, where they can modulate immune responses and prevent neuronal damage [30]. Moreover, in inflammatory bowel disease, nanoparticles and liposomes have been used to deliver therapeutic agents directly to the gastrointestinal tract, where they can reduce inflammation and promote tissue healing without affecting other organs [31]. So, nanotechnology holds great promise in revolutionizing the treatment of autoimmune diseases by enabling targeted, controlled, and localized drug delivery. The development of biodegradable and stimuli-responsive nanomaterials offers an exciting opportunity to improve the precision and efficacy of treatments while minimizing side effects and systemic toxicity. Figure 3 highlights the diverse therapeutic potential of nanomedicine in managing autoimmune diseases, transitioning from traditional immunosuppressive strategies to the induction of immune tolerance [32]. Nanotechnology-based approaches enable precise delivery of immunosuppressive agents, minimizing systemic side effects and enhancing efficacy at the disease site. Beyond conventional therapies, nanomaterials are being developed to promote immune tolerance by modulating immune responses to restore self-recognition and prevent tissue damage. These nanocarriers, including nanoparticles and hydrogels, can be tailored to deliver antigens, cytokines, or regulatory agents in a controlled manner, reprogramming the immune system to distinguish between self and non-self. This advancement represents a paradigm shift, offering targeted and potentially curative options for autoimmune disease management.

## 2. Stimuli-Responsive Nanomaterials for Targeted Therapy

Stimuli-responsive nanomaterials have gained significant attention as innovative platforms for targeted drug delivery, particularly in addressing complex diseases such as cancer, autoimmune disorders, and infections. These nanomaterials exhibit the ability to undergo specific and controlled physicochemical transformations in response to external or internal environmental cues, such as pH variations, temperature fluctuations, redox gradients, or the presence of certain enzymes. This adaptability allows for the precise modulation of drug release at the desired site of action, minimizing systemic side effects and enhancing therapeutic efficacy. This section provides an in-depth exploration of various stimuli-responsive systems, elucidating the underlying mechanisms by which these nanomaterials respond to distinct triggers. Furthermore, it highlights their applications in advancing drug delivery technologies, enabling precise and effective treatment strategies tailored to individual pathological conditions.

### 2.1. Types of Stimuli and Responsiveness of Nanomaterial Mechanisms

Stimuli-responsive nanomaterials are designed to respond to environmental changes within the body. By harnessing these changes, such materials can release their therapeutic payloads in a controlled and targeted manner, optimizing treatment efficacy and minimizing side effects. Below, we discuss the key types of stimuli that influence the responsiveness of nanomaterials.

#### 2.1.1. pH-Responsive Nanomaterial Systems

pH is one of the most commonly exploited stimuli for the development of drug delivery systems. The human body exhibits different pH levels in various compartments, such as in the bloodstream (around pH 7.4), acidic tumors (pH 5.5–6.5), and the inflamed microenvironment in autoimmune diseases (which can also be acidic). pH-responsive nanomaterials are designed to release their drug payload in response to these pH changes, thereby targeting specific tissues or disease sites.

Mechanism of Action: pH-responsive systems are typically based on polymers that contain pH-sensitive groups, such as carboxyl (-COOH) or amino (-NH2) groups, which undergo ionization depending on the surrounding pH. For example, when these materials are in a more acidic environment (such as that found in tumors or inflamed tissues), the ionizable groups on the polymer become protonated, leading to a change in the material’s solubility or structure. This results in the rupture or swelling of the nanocarrier and the subsequent release of the encapsulated drug [33].

**Figure 4:**
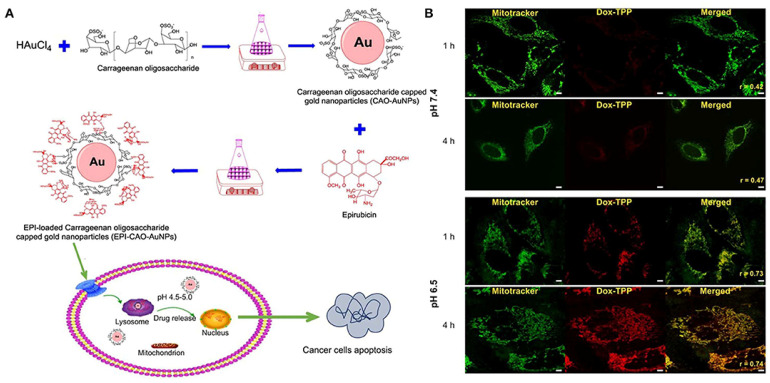
pH-responsive drug delivery systems. (**A**) Epirubicin-loaded gold nanoparticles capped with carrageenan oligosaccharides demonstrate pH-dependent behavior. (**B**) The release of the drug from Dox-TPP nanoparticles was significantly enhanced in the acidic intracellular environment (pH 6.5), while minimal release occurred at a neutral pH (7.5) after 4 h. Adapted with permission from Ref. [35].

Applications: pH-responsive systems are highly useful for treating localized inflammation, such as in autoimmune diseases like rheumatoid arthritis, where the inflamed tissue can be targeted more effectively. Nanoparticles made of materials such as polyacrylic acid (PAA), poly(ethylene glycol) (PEG), or polylactic-co-glycolic acid (PLGA) can be functionalized to release their drug payload at sites where the pH is lower than normal, ensuring targeted delivery [34]. Figure 4 illustrates the functionality of pH-responsive drug delivery systems designed for targeted release within cellular environments [35]. In part (A), epirubicin-loaded gold nanoparticles, capped with carrageenan oligosaccharides, exhibit a tailored response to pH variations. These nanoparticles remain stable under neutral conditions but are activated in acidic microenvironments, such as those found in tumor cells or inflamed tissues. Part (B) demonstrates the pH-sensitive release profile of Dox-TPP nanoparticles, where significant drug release is observed at an acidic pH of 6.5, mimicking intracellular compartments, while minimal release occurs at a neutral pH of 7.5. This targeted release mechanism minimizes off-target effects and enhances therapeutic efficacy, making such systems promising for precision medicine applications in cancer and other diseases.

#### 2.1.2. Temperature-Responsive Nanomaterial Systems

Temperature-responsive nanomaterials take advantage of the temperature fluctuations that occur in different parts of the body, such as during inflammation, fever, or at certain disease sites. In addition, localized heating can be applied externally to increase the temperature at the target site, enhancing the release of therapeutic agents. These systems are typically based on polymers or copolymers that undergo reversible phase transitions with temperature changes. Temperature-responsive materials often contain hydrophilic and hydrophobic segments that form a gel or polymer network. At a specific “lower critical solution temperature” (LCST), these materials undergo a phase transition, shifting from a swollen (hydrophilic) to a collapsed (hydrophobic) state. This transition alters the solubility and swelling behavior of the material, facilitating the release of the encapsulated drug. For example, poly(N-isopropylacrylamide) (PNIPAAm) is a commonly used temperature-sensitive polymer that exhibits an LCST around 32 °C. At this temperature, the material changes its conformation, leading to drug release [36]. Temperature-sensitive nanomaterials are ideal for drug delivery systems in conditions such as rheumatoid arthritis, where localized temperature changes can be utilized for controlled release. Additionally, externally applied heat (e.g., via infrared radiation) can increase the temperature at the target site to trigger drug release from these systems, making them highly versatile for localized therapy.

#### 2.1.3. Redox-Responsive Nanomaterial Systems

The redox state of a particular environment can also serve as a trigger for nanomaterial-based drug delivery. The body’s tissues, particularly in certain disease states, have distinct redox potentials that differ significantly from healthy tissues. For example, inflamed or cancerous tissues often have an elevated concentration of reactive oxygen species (ROS) or reduced levels of antioxidants. These variations in redox balance provide an ideal opportunity to design nanomaterials that release their therapeutic payload in response to changes in the oxidative state. Redox-responsive drug delivery systems generally rely on the incorporation of disulfide bonds or other redox-sensitive linkers in the nanomaterial. The reduction of disulfide bonds by intracellular reducing agents such as glutathione (GSH) or ROS leads to the cleavage of the linker, which results in the release of the drug. For example, the use of disulfide-bridged polymeric nanoparticles enables drug release upon exposure to high GSH concentrations found in tumors or inflamed tissues [37]. Redox-responsive systems are particularly useful for targeted drug delivery to tumors, autoimmune disease sites, and areas with chronic inflammation. In rheumatoid arthritis, where oxidative stress plays a key role in disease progression, redox-sensitive systems can be designed to release anti-inflammatory drugs specifically at the inflamed joints, minimizing systemic side effects and maximizing therapeutic efficacy [38].

#### 2.1.4. Enzyme-Responsive Nanomaterial Systems

Enzymes are biological catalysts that play key roles in various physiological processes. The expression of specific enzymes is often dysregulated in certain disease conditions, including autoimmune diseases, cancer, and infections. Enzyme-responsive nanomaterials are engineered to exploit the presence of disease-specific enzymes, leading to the targeted release of drugs at the disease site. Enzyme-responsive drug delivery systems typically contain substrates that are selectively cleaved by overexpressed enzymes in the pathological environment. For example, certain enzymes, such as matrix metalloproteinases (MMPs), are known to be upregulated in inflammatory conditions and cancer. Nanomaterials can be functionalized with peptide sequences that are cleaved by these enzymes, leading to the release of the drug payload. Other enzyme-responsive systems rely on the enzymatic cleavage of prodrugs, which become activated upon exposure to specific enzymes [39]. Enzyme-responsive drug delivery systems are highly promising for applications in autoimmune diseases like rheumatoid arthritis, where specific enzymes such as MMPs and collagenases are overexpressed in the inflamed synovium. By designing nanocarriers that respond to these enzymes, drugs can be precisely delivered to the site of inflammation, ensuring therapeutic efficacy while reducing systemic toxicity [40]. Stimuli-responsive nanomaterials offer highly dynamic and adaptable platforms for targeted drug delivery. By exploiting environmental triggers such as pH, temperature, redox potential, and enzymatic activity, these materials can provide controlled, localized drug release, ensuring enhanced therapeutic effects with reduced side effects. The development of such systems represents a promising strategy in the treatment of autoimmune diseases, cancer, and other pathologies requiring precise drug targeting and controlled release. Table 1 compares various stimuli-responsive mechanisms in nanomaterial drug delivery systems, highlighting the unique advantages and challenges of pH-responsive, temperature-responsive, redox-responsive, and enzyme-responsive systems. pH-responsive systems are particularly useful for targeting inflamed or tumor tissues, which often have acidic microenvironments, allowing for localized and controlled drug release at the disease site. Temperature-responsive systems, on the other hand, provide non-invasive mechanisms where drug release is triggered by temperature variations, making them suitable for localized heating or conditions like fever in autoimmune diseases. Redox-responsive systems exploit the oxidative stress prevalent in inflammatory diseases, such as rheumatoid arthritis, to release drugs precisely where needed. However, controlling the release in such systems can be challenging due to variations in the redox environment. Enzyme-responsive systems, tailored to recognize and respond to overexpressed enzymes in specific disease states, provide another layer of specificity in drug delivery, particularly for targeting tissue degradation enzymes in diseases like rheumatoid arthritis. While each system offers distinct advantages, such as enhanced specificity and reduced systemic side effects, they also present challenges related to the complexity of triggering mechanisms and the potential for variability in different patients.

### 2.2. Controlled Release Mechanisms for Autoimmune Diseases

Controlled release mechanisms are fundamental to optimizing the therapeutic efficacy of drug delivery systems, particularly in the treatment of chronic diseases such as autoimmune disorders, cancer, and infections. These mechanisms ensure that drugs are delivered at a steady rate over time, maintaining effective therapeutic concentrations while minimizing side effects. In the context of stimuli-responsive nanomaterials, controlled release can be precisely regulated in response to environmental changes or external triggers, offering significant advantages in terms of targeted therapy and patient compliance.

#### 2.2.1. Types of Controlled Release Mechanisms for Autoimmune Diseases

Several strategies can be employed to control the release of drugs from nanomaterials. These include physical, chemical, and environmental stimuli-driven mechanisms, which allow for the precise modulation of drug release at the target site.

##### Diffusion-Controlled Release for Autoimmune Diseases

Diffusion-controlled release is one of the simplest and most widely used mechanisms for controlled drug delivery. In this system, the drug diffuses out of the nanocarrier through a concentration gradient. The rate of drug release depends on the diffusion properties of both the drug and the carrier matrix. In diffusion-controlled systems, the drug is typically encapsulated in a matrix or reservoir, and its release occurs as the drug molecules move from the high-concentration core to the lower-concentration surroundings. The release rate is governed by factors such as the size of the nanocarrier, the molecular weight of the drug, and the permeability of the carrier material. For example, polymeric nanoparticles, liposomes, or hydrogels can release encapsulated drugs through passive diffusion when the environmental conditions are stable [41]. This mechanism is often used for sustained release of drugs over extended periods, making it suitable for chronic diseases where continuous drug delivery is required, such as in autoimmune diseases like rheumatoid arthritis. By modifying the properties of the nanomaterials (e.g., by increasing the crosslinking density of hydrogels), the release rate can be finely tuned to meet therapeutic needs.

##### Swelling-Controlled Release for Autoimmune Diseases

Swelling-controlled release mechanisms are typically used in hydrogels and other porous nanomaterials. In this system, the nanocarrier swells in response to environmental factors such as pH, temperature, or ionic strength, leading to the release of the drug. In swelling-controlled systems, drugs are encapsulated in a carrier matrix, which swells or expands upon exposure to specific environmental stimuli. The degree of swelling affects the release rate of the drug, with a higher degree of swelling leading to faster release. This type of system is particularly useful for drugs that require a controlled, gradual release over time, such as anti-inflammatory drugs in the treatment of autoimmune diseases. Swelling may be triggered by a change in pH (acidic environments) or by temperature shifts, which induce conformational changes in the polymer network [42]. Swelling-controlled release systems are often employed in the delivery of anti-cancer drugs or biologics in specific regions of inflammation, where changes in pH or temperature can trigger drug release. For example, hydrogels with temperature-sensitive properties can swell and release the encapsulated drug when exposed to localized heating or temperature fluctuations associated with inflammation.

##### Degradation-Controlled Release for Autoimmune Diseases

Degradation-controlled release is a mechanism where the nanomaterial itself degrades or dissolves over time, gradually releasing the drug in the process. This is a key feature of many biodegradable nanocarriers, including polymeric nanoparticles, micelles, and hydrogels. In degradation-controlled release, the nanocarrier is designed to degrade in a controlled manner under specific conditions, such as in response to changes in pH, temperature, or the presence of enzymes. As the carrier degrades, it releases the encapsulated drug in a sustained manner. The degradation rate of the material can be engineered to match the desired release profile of the drug. For example, PLGA-based nanoparticles can degrade through hydrolysis, releasing the drug as the polymer breaks down into biocompatible products such as lactic acid and glycolic acid [43]. Degradation-controlled systems are particularly useful in the delivery of anti-inflammatory or immunosuppressive drugs for the treatment of autoimmune diseases. These systems allow for continuous drug release without the need for repeated administrations. For example, biodegradable nanoparticles or micelles that degrade in response to the acidic microenvironment of an inflamed tissue can provide localized and sustained drug release for the treatment of conditions like rheumatoid arthritis.

##### Stimuli-Responsive Release for Autoimmune Diseases

Stimuli-responsive release mechanisms are based on the concept of using external or internal environmental stimuli, such as pH, temperature, light, magnetic fields, or enzymatic activity, to trigger the release of the drug. These systems are designed to respond to specific signals at the target site, ensuring that the drug is only released when it reaches the desired location or under certain conditions. Stimuli-responsive nanomaterials often include smart polymers or bioactive agents that can undergo physical or chemical changes in response to external or internal stimuli. For example, pH-sensitive nanomaterials will undergo a structural change when exposed to an acidic environment, leading to the release of the encapsulated drug. Similarly, temperature-sensitive carriers can release their payload when exposed to elevated temperatures, such as in a localized inflammatory region. Enzyme-sensitive carriers can degrade or cleave in the presence of specific enzymes, releasing the drug only when those enzymes are present at the target site [44]. Stimuli-responsive release mechanisms are particularly valuable for treating diseases where localized drug delivery is crucial, such as in cancer and autoimmune diseases. For example, a nanocarrier designed to respond to the elevated oxidative stress or enzymatic activity in an inflamed joint would release the therapeutic drug only at the site of inflammation, reducing systemic toxicity and enhancing therapeutic efficacy.

##### Controlled Release via Diffusion and Degradation Combined for Autoimmune Diseases

Some advanced nanomaterial-based drug delivery systems combine diffusion-controlled and degradation-controlled mechanisms to provide a more precise and sustained drug release. In these systems, the drug release occurs via both diffusion through the matrix and degradation of the nanocarrier, providing a dual mechanism for controlled release. In these combined systems, the initial drug release occurs via diffusion, followed by sustained release as the nanocarrier undergoes gradual degradation. The dual mechanism can be finely tuned by adjusting the material properties, such as the molecular weight of the polymer, the crosslinking density, and the size of the nanoparticles, to achieve the desired release profile. For instance, PLGA nanoparticles often combine both diffusion and degradation mechanisms for sustained release over a prolonged period [45]. Such systems are ideal for chronic conditions that require long-term drug delivery, like autoimmune diseases. These systems ensure that the drug is released in a controlled manner over an extended period, which helps maintain therapeutic concentrations without requiring frequent dosing.

#### 2.2.2. Modulating Release Rates for Autoimmune Diseases

To further optimize controlled release, the rate of drug release can be modulated by altering the physical or chemical properties of the nanocarriers. Factors such as polymer composition, particle size, surface charge, and the presence of functional groups can all influence the release profile of the drug.

➢ Polymer Composition: The choice of polymer used in the nanocarrier can significantly impact the release rate. For instance, hydrophobic polymers will release drugs more slowly compared to hydrophilic ones, as hydrophobic drugs tend to interact more strongly with the polymer matrix, delaying their release [46].
➢ Particle Size: Smaller nanoparticles tend to have a higher surface area-to-volume ratio, which can lead to faster release. By controlling the particle size, it is possible to fine-tune the drug release profile to achieve the desired therapeutic effect.
➢ Surface Charge: Nanocarriers with different surface charges can interact differently with biological membranes and cells. Altering the surface charge can help improve drug retention and modify the release profile, particularly for drugs that need to be delivered to specific cells or tissues [47].

Controlled release mechanisms are essential for ensuring that drugs are delivered at optimal rates and concentrations, minimizing side effects and maximizing therapeutic efficacy. Different approaches, including diffusion-controlled, swelling-controlled, degradation-controlled, and stimuli-responsive release systems, offer distinct advantages depending on the therapeutic target. By combining these mechanisms or modulating their parameters, nanomaterials can be tailored to provide precise, sustained drug release for the treatment of chronic diseases like autoimmune disorders, cancer, and infections.

## 3. Applications in Autoimmune Disease Treatment

Autoimmune diseases, where the immune system mistakenly attacks the body’s own tissues, pose significant challenges to treatment due to their chronic nature and varied manifestations. Traditional treatments often involve immunosuppressive drugs, but these can lead to systemic side effects. The development of nanomaterial-based drug delivery systems offers a promising solution by allowing for targeted and controlled delivery of therapeutics directly to inflamed tissues, improving treatment efficacy and minimizing adverse effects. This section discusses the application of nanomaterials in the treatment of two major autoimmune diseases: rheumatoid arthritis (RA) and multiple sclerosis (MS).

### 3.1. Rheumatoid Arthritis (RA)

Rheumatoid arthritis (RA) is a chronic inflammatory autoimmune disorder characterized by the immune system attacking the synovial joints, leading to pain, swelling, and, if left untreated, joint destruction. Current treatments for RA include disease-modifying antirheumatic drugs (DMARDs), biologics, and nonsteroidal anti-inflammatory drugs (NSAIDs). However, these therapies often require systemic administration and may cause significant side effects. Nanomaterial-based drug delivery systems hold the potential to improve the targeting and effectiveness of RA treatments by delivering drugs directly to the inflamed joints, thereby reducing systemic toxicity and enhancing local drug concentration.

**Nanomaterial-Based Strategies for RA Treatment**

➢ pH-Responsive Nanoparticles: RA is associated with localized inflammation and a decrease in pH levels in the synovial fluid. pH-responsive nanoparticles can be engineered to release drugs in response to the acidic environment of inflamed joints. For example, nanoparticles made from polyacrylic acid (PAA) or other pH-sensitive polymers can encapsulate anti-inflammatory drugs, such as methotrexate or corticosteroids, and release them when exposed to the acidic pH in the inflamed synovial fluid [48,49].
➢ Nanocarriers for Targeted Drug Delivery: Liposomes and polymeric nanoparticles functionalized with targeting ligands (such as antibodies or peptides) can be used to specifically target the inflamed joints, thus ensuring that drugs are delivered directly to the affected area. This targeted delivery minimizes systemic side effects, such as immunosuppression or gastrointestinal issues, associated with traditional RA therapies [50].
➢ Intra-Articular Nanocarriers: Direct delivery of nanoparticles into the joint space, known as intra-articular delivery, has been explored as a strategy for RA treatment. These nanoparticles can encapsulate anti-inflammatory agents or biologics and provide sustained release at the site of inflammation. For instance, PLGA-based nanoparticles have been shown to improve the therapeutic effect of glucocorticoids and biologics when administered directly into the joint [51].
➢ Gene Therapy Nanocarriers: Gene therapy approaches using nanomaterials are being investigated for RA treatment. Nanocarriers can deliver anti-inflammatory cytokine genes or small interfering RNA (siRNA) targeting specific pro-inflammatory genes (such as TNF-α or IL-1β) in the inflamed joints. This approach allows for a more precise and long-lasting therapeutic effect compared to traditional biologics [52].

### 3.2. Multiple Sclerosis (MS)

Multiple sclerosis (MS) is a chronic autoimmune disease where the immune system attacks the central nervous system (CNS), leading to neuroinflammation, demyelination, and progressive neurological disability. MS treatment typically involves immunomodulatory therapies, including corticosteroids, interferons, and monoclonal antibodies. However, these treatments are often associated with side effects and may not effectively target the site of inflammation in the CNS. Nanotechnology offers innovative solutions for MS treatment by facilitating targeted drug delivery across the blood–brain barrier (BBB) and reducing systemic exposure to drugs.

Nanomaterial-Based Strategies for MS Treatment

➢ Blood–Brain Barrier (BBB) Penetration: One of the biggest challenges in MS treatment is the efficient delivery of drugs to the CNS. Nanoparticles, particularly those functionalized with specific ligands (such as transferrin or apolipoprotein E), have been shown to improve BBB penetration. These nanoparticles can be designed to cross the BBB and deliver anti-inflammatory or immunosuppressive drugs directly to the brain and spinal cord [53].
➢ Nanoparticles for Immunomodulation: In MS, T cell-mediated immune responses play a key role in the progression of the disease. Nanoparticles can be designed to modulate immune responses, either by delivering immunosuppressive drugs or by directly targeting immune cells. For example, lipid-based nanoparticles loaded with glucocorticoids or siRNA targeting pro-inflammatory cytokines can be used to suppress the autoimmune response in MS without affecting the overall immune system [54].
➢ Encapsulation of Disease-Modifying Drugs: Disease-modifying drugs (DMDs), such as glatiramer acetate and interferon beta, are commonly used to treat MS, but their use is often limited by side effects and inconsistent efficacy. Nanocarriers, such as liposomes or solid lipid nanoparticles, can encapsulate these drugs and provide controlled, sustained release, enhancing the drug’s bioavailability and therapeutic efficacy. Furthermore, these carriers can target the drug directly to inflamed regions of the CNS, ensuring a more focused and effective treatment approach [55].
➢ Nanoparticle-Based Vaccines for MS: Vaccine-based therapies for MS are under investigation, aiming to modulate the immune system’s response to myelin proteins and prevent or slow down disease progression. Nanoparticles, particularly those made from biocompatible materials such as chitosan or poly(lactic-co-glycolic acid), can be used as carriers for myelin-derived antigens, presenting them to the immune system in a controlled manner to promote tolerance and prevent autoimmune attacks on the CNS [56].
➢ Exosome-Based Nanotherapy: Exosomes, small vesicles secreted by cells, have been explored as a potential therapeutic platform for MS. Exosomes can be engineered to deliver anti-inflammatory agents or even genetic material directly to immune cells or neurons in the CNS. They have the advantage of being naturally capable of crossing the BBB and offering low immunogenicity, making them an ideal candidate for MS treatment [57].

Nanomaterial-based drug delivery systems offer significant promise for the treatment of autoimmune diseases such as rheumatoid arthritis and multiple sclerosis. These systems can enhance the precision of drug delivery, ensure sustained release, and minimize systemic side effects. For RA, nanotechnology enables targeted delivery to inflamed joints, while for MS, it addresses the challenge of delivering drugs across the blood–brain barrier. By using pH-sensitive, temperature-responsive, or immunomodulatory nanoparticles, these novel approaches can greatly improve the management of autoimmune diseases, offering more effective, localized, and less toxic therapeutic options.

Table 2 presents a comparative analysis of the applications of nanomaterials in treating various autoimmune diseases. It highlights the types of nanomaterials used, the therapeutic targets, the key advantages of these systems, and the challenges that remain in their application. For example, in rheumatoid arthritis (RA), polymeric nanoparticles and liposomes are effective for targeted drug delivery to inflamed joints, but polymer toxicity and liposome stability are ongoing concerns. Similarly, in multiple sclerosis (MS), hydrogels and pH-responsive systems offer controlled release of immune modulators, but challenges like crossing the blood–brain barrier persist. Additionally, nanomaterials are showing promise in diseases such as lupus, inflammatory bowel disease (IBD), and psoriasis, providing targeted delivery of immunosuppressive agents and cytokine inhibitors with reduced systemic side effects [58]. Despite their potential, issues such as cytotoxicity, immunogenicity, and formulation complexities need to be addressed for their clinical translation.

### 3.3. Inflammatory Bowel Disease (IBD)

Inflammatory bowel disease (IBD), which includes conditions such as Crohn’s disease and ulcerative colitis, is a chronic autoimmune disorder characterized by inflammation of the gastrointestinal tract. The disease is caused by an overactive immune response to the gut microbiota, leading to damage in the intestinal lining. Conventional treatments for IBD involve corticosteroids, immunosuppressants, and biologic agents like anti-TNF therapies. However, these treatments often result in systemic side effects and may not be effective in all patients. Nanotechnology offers an innovative approach to address these challenges by enabling targeted drug delivery directly to the inflamed gut, improving the localized therapeutic effects while minimizing systemic exposure.

Nanomaterial-Based Strategies for IBD Treatment

➢ pH-Responsive Nanoparticles: The pH of the gastrointestinal tract varies along its length, with the stomach being acidic (pH 1–3) and the colon being more alkaline (pH 7–8). pH-responsive nanoparticles can be designed to release their payload at specific locations within the gut. For instance, nanoparticles made from materials such as Eudragit or chitosan are designed to remain stable in the acidic environment of the stomach and only release drugs when they reach the more neutral or basic environment of the colon, where inflammation in IBD is most prominent [65]. This targeted release reduces drug degradation in the stomach and enhances the therapeutic effects at the site of inflammation.
➢ Targeted Delivery via Ligand-Functionalized Nanoparticles: Ligand-functionalized nanoparticles are used to target specific receptors on the inflamed tissues in the gut. For example, nanoparticles coated with antibodies against the inflammatory cytokine TNF-α or adhesion molecules like integrins can selectively bind to the inflamed areas of the intestines. This ensures that therapeutic agents, such as corticosteroids or biologics, are delivered directly to the site of inflammation, enhancing efficacy and reducing systemic side effects [66].
➢ Polymeric Micelles for Drug Encapsulation: Polymeric micelles, formed from amphiphilic copolymers, can encapsulate hydrophobic drugs and protect them from degradation in the gastrointestinal tract. These micelles are small enough to penetrate the intestinal mucosa, providing efficient delivery to the target area. For example, micelles loaded with anti-inflammatory drugs such as methotrexate or 5-aminosalicylic acid (5-ASA) have been shown to reduce inflammation in animal models of IBD [67,68]. The release of the drug is often controlled by pH-sensitive linkages, which degrade in the more alkaline environment of the intestines.
➢ Nanoparticles for Gene Therapy: Gene therapy using nanocarriers holds promise in treating IBD by delivering genetic material that can modulate the immune response. Nanoparticles can be used to deliver small interfering RNA (siRNA) or messenger RNA (mRNA) targeting inflammatory cytokines such as TNF-α or interleukin-1β (IL-1β), thereby reducing inflammation. This approach may offer a more targeted and long-term treatment for patients with chronic IBD [69].

**Figure 5:**
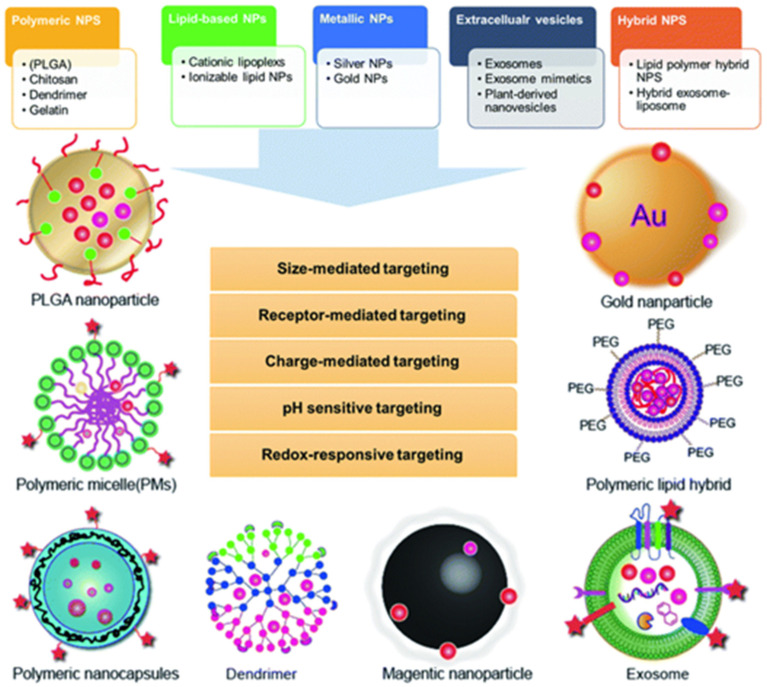
Promising nanocarriers for targeted drug delivery in inflammatory bowel disease (IBD). Adapted with permission from ref. [70]. Copyright 2022 Royal Society of Chemistry.

Figure 5 showcases the various types of nanocarriers that hold significant promise for targeted drug delivery in the treatment of inflammatory bowel disease (IBD) [70]. These nanocarriers, including nanoparticles, liposomes, and micelles, are engineered to specifically target the inflamed gastrointestinal tract, enhancing drug bioavailability at the site of disease. By utilizing features such as surface functionalization with targeting ligands and responsiveness to the unique pH or enzymatic conditions present in the IBD-affected areas, these nanocarriers can improve therapeutic outcomes while minimizing systemic side effects. The ability to encapsulate and deliver anti-inflammatory agents, biologics, or other therapeutic molecules with high precision offers a new avenue for IBD management, potentially reducing the need for systemic therapies and providing more effective, localized treatment options.

### 3.4. Case Studies of Stimuli-Responsive Drug Delivery in Autoimmune Diseases

Stimuli-responsive drug delivery systems have been widely researched and applied in the treatment of autoimmune diseases due to their ability to release drugs in a controlled manner in response to environmental signals. This section discusses key case studies where stimuli-responsive nanomaterials have been successfully utilized for targeted therapy in autoimmune diseases.

Case Study 1: pH-Responsive Nanoparticles in Rheumatoid Arthritis (RA)

A study by Zhao et al. demonstrated the use of pH-sensitive nanoparticles for the targeted delivery of methotrexate (MTX) to inflamed joints in rheumatoid arthritis. The nanoparticles, made from polyacrylic acid (PAA), were designed to remain stable at neutral pH but undergo rapid degradation and release the drug at the acidic pH found in inflamed synovial fluid. In vivo studies showed that these nanoparticles significantly reduced inflammation and joint damage in rat models of RA, while minimizing the systemic side effects commonly associated with oral MTX therapy [71].

Findings:

- pH-sensitive nanoparticles efficiently delivered methotrexate to inflamed joints.
- Significant reduction in joint inflammation and damage.
- Minimized systemic side effects compared to free methotrexate.

Case Study 2: Temperature-Responsive Nanogels in Multiple Sclerosis (MS)

In multiple sclerosis, the goal is to reduce inflammation and immune cell infiltration into the central nervous system (CNS). A study by Shobeirean et al. investigated temperature-sensitive nanogels for the controlled release of glatiramer acetate, a drug used to treat MS. These nanogels were designed to release their therapeutic cargo in response to the elevated temperature typically associated with inflammation. Upon injection into experimental animals with MS, the nanogels effectively localized the drug at the site of inflammation in the CNS and improved the clinical symptoms of the disease [72].

Findings:

- Temperature-sensitive nanogels enhanced the release of glatiramer acetate at sites of inflammation.
- Improved targeting and therapeutic efficacy in the CNS.
- Reduced systemic drug exposure and side effects.

Case Study 3: Enzyme-Responsive Nanoparticles in Inflammatory Bowel Disease (IBD)

A study by Tang et al. explored enzyme-responsive nanoparticles for the treatment of inflammatory bowel disease (IBD). These nanoparticles were designed to release their therapeutic cargo in response to specific enzymes overexpressed in the inflamed intestinal tissues, such as colonic esterases. The nanoparticles were loaded with the anti-inflammatory drug 5-aminosalicylic acid (5-ASA) and demonstrated enhanced drug release in inflamed sections of the colon. In animal models of IBD, the enzyme-responsive nanoparticles significantly reduced inflammation and promoted healing of the intestinal mucosa [73].

Findings:

- Enzyme-responsive nanoparticles provided targeted drug delivery to inflamed areas of the gut.
- Significant reduction in intestinal inflammation in IBD models.
- Enhanced therapeutic effect with minimal side effects.

Case Study 4: Redox-Responsive Nanocarriers for Systemic Lupus Erythematosus (SLE)

Systemic lupus erythematosus (SLE) is characterized by oxidative stress and the production of reactive oxygen species (ROS) in affected tissues. A study by Rajes et al. developed redox-responsive nanocarriers for the targeted delivery of dexamethasone to sites of inflammation in SLE. These nanocarriers were designed to release the drug in response to the high levels of ROS present in inflamed tissues. In vitro studies showed that the nanocarriers had enhanced drug release in the presence of ROS, and in vivo studies in SLE mouse models demonstrated significant improvement in symptoms such as skin lesions and joint inflammation [74].

Findings:

- Redox-responsive nanocarriers effectively delivered dexamethasone to inflamed tissues.
- Significant reduction in inflammation and clinical symptoms of SLE.
- Reduced systemic side effects compared to conventional therapy.

Stimuli-responsive drug delivery systems are revolutionizing the treatment of autoimmune diseases by enabling targeted and controlled drug release in response to specific environmental signals. These systems improve the precision and efficacy of treatments, particularly in diseases like rheumatoid arthritis, multiple sclerosis, inflammatory bowel disease, and systemic lupus erythematosus. The case studies discussed demonstrate the potential of pH-, temperature-, enzyme-, and redox-responsive nanomaterials to enhance therapeutic outcomes while minimizing side effects. Continued research in this field promises to further optimize the use of nanotechnology in autoimmune disease therapy, paving the way for more personalized and effective treatments.

## 4. Challenges in Clinical Translation

The translation of nanomaterial-based drug delivery systems from the laboratory to clinical applications presents several challenges [75]. Despite the promising results observed in preclinical studies, the clinical adoption of these technologies faces significant hurdles related to biocompatibility, immune response, manufacturing, and scalability. This section will discuss these challenges in more detail, focusing on biocompatibility and immune response as well as the difficulties in large-scale manufacturing of nanomaterials for drug delivery.

### 4.1. Biocompatibility and Immune Response

One of the key challenges in the clinical translation of nanomaterial-based drug delivery systems is ensuring their biocompatibility and minimizing any adverse immune responses. Nanoparticles, when introduced into the body, interact with various cells and tissues, and the immune system may recognize them as foreign objects. These interactions can lead to undesirable immune responses, such as inflammation, cytotoxicity, and in some cases, the activation of the complement system or the formation of anti-nanoparticle antibodies. The body’s immune system may clear the nanoparticles prematurely, reducing their therapeutic efficacy [76].

Biocompatibility of Nanomaterials

➢ Material Choice: The choice of material for designing nanoparticles plays a crucial role in their biocompatibility. Biodegradable and biocompatible materials, such as poly(lactic-co-glycolic acid) (PLGA), chitosan, and lipids, are commonly used for drug delivery applications because they are metabolized or excreted from the body after fulfilling their therapeutic role. However, the breakdown products of some nanomaterials may induce toxicity or cause inflammation if they accumulate in tissues over time [77].
➢ Surface Functionalization: The surface properties of nanoparticles, including size, charge, and hydrophilicity, can significantly influence their interaction with the immune system. For example, nanoparticles with a high positive charge may aggregate in vivo, leading to an immune response, while neutral or slightly negative charges are generally associated with reduced immune activation. Surface coatings, such as polyethylene glycol (PEG), can also help “stealth” the nanoparticles, reducing their recognition by the immune system and prolonging their circulation time in the bloodstream [78].
➢ Toxicity Concerns: Nanoparticles, particularly those made from non-degradable materials, may accumulate in organs such as the liver, spleen, or lungs, leading to long-term toxicity. Non-toxic degradation products are essential to ensure that nanoparticles do not pose long-term risks to health. Studies on the long-term fate of nanoparticles in the body are still needed to better understand their potential accumulation and effects in tissues and organs [79].
➢ Inflammation and Complement Activation: When nanoparticles are administered into the body, they can interact with plasma proteins, leading to complement activation and subsequent immune responses. This can result in systemic inflammation or anaphylaxis in some cases [80]. Therefore, careful design of nanoparticle surface properties and the use of biocompatible materials are critical for minimizing unwanted immune responses.
➢ Cellular Uptake and Biodistribution: Nanoparticles’ ability to be taken up by cells also depends on their size and surface characteristics. Nanoparticles that are too large or that carry certain surface charges may be less effective at reaching their target tissues or organs. Additionally, the biodistribution of nanoparticles can lead to off-target effects, where drugs are delivered to unintended organs, potentially causing harm or inefficiency in treatment [81].

### 4.2. Manufacturing and Scalability

The successful commercialization of nanomaterial-based drug delivery systems hinges on the ability to manufacture them at large scales while maintaining their quality, safety, and efficacy. The current challenges in manufacturing and scalability are multifaceted, ranging from technical limitations in production methods to regulatory hurdles in ensuring the consistency and safety of large batches of nanomaterials.

Challenges in Manufacturing Nanomaterials

➢ Nanoparticle Production Methods: There are several methods for producing nanoparticles, such as solvent evaporation, high-pressure homogenization, and nanoprecipitation. However, these methods often face limitations in terms of reproducibility, scalability, and control over the properties of the nanoparticles, such as size, morphology, and surface charge. For example, controlling the size distribution of nanoparticles during synthesis is crucial for ensuring consistent drug release profiles, but this can be difficult at larger scales. Additionally, batch-to-batch variability can occur, leading to inconsistency in product quality and therapeutic efficacy [82].
➢ High-Throughput Production: One of the main challenges in scaling up nanoparticle production is achieving high throughput while maintaining uniform quality. Traditional production methods often require manual intervention or lengthy procedures, which are not easily scalable. More advanced methods, such as microfluidic devices and automated production lines, are being explored to overcome these challenges. However, these technologies still need to be optimized for large-scale manufacturing [83].
➢ Quality Control and Standardization: To meet regulatory standards, it is essential to establish stringent quality control measures for nanoparticle production. This includes ensuring consistent particle size, surface charge, drug encapsulation efficiency, and release profiles. However, due to the complex and dynamic nature of nanoparticles, establishing standardized protocols for their production remains a significant challenge. Nanoparticles often exhibit variability in their size, shape, and surface properties depending on the manufacturing conditions, and achieving uniformity across large batches is critical for ensuring consistent therapeutic outcomes [84].
➢ Cost and Commercial Viability: The production of nanomaterials for drug delivery can be costly due to the need for specialized equipment, high-quality raw materials, and time-consuming processing steps. Reducing production costs while maintaining high quality and scalability is a critical factor in the commercialization of nanomaterial-based therapies. For example, liposomes and polymeric nanoparticles often require expensive raw materials and complex synthesis methods. Moving forward, there is a need for more cost-effective production methods that can lower the cost of goods and make these therapies commercially viable [85].
➢ Regulatory and Safety Concerns: Regulatory agencies such as the FDA and EMA require rigorous testing and documentation before approving nanomaterial-based drug delivery systems for clinical use. Nanoparticles must undergo a variety of tests, including biocompatibility, stability, pharmacokinetics, and toxicity evaluations, which can be time-consuming and expensive. Regulatory guidelines for the manufacture and use of nanomaterials are still evolving, and the lack of clear, standardized procedures makes it challenging for manufacturers to navigate the approval process [86].

Technological Advances and Potential Solutions

Several advancements in nanomaterial manufacturing technologies may help address scalability and manufacturing challenges:

- Microfluidics: Microfluidic devices offer precise control over nanoparticle size, surface properties, and drug encapsulation, which can be essential for high-throughput, reproducible production at large scales. The use of microfluidics can significantly enhance the consistency and uniformity of nanoparticle production [87].
- Continuous Flow Production: Continuous flow systems, as opposed to batch production, can allow for scalable and efficient nanoparticle synthesis. This method could provide more control over the reaction conditions, leading to better reproducibility and reduced production time [88].
- Green Manufacturing Approaches: Eco-friendly production methods using biocompatible solvents, renewable resources, and energy-efficient processes are being explored to reduce the environmental impact and cost of manufacturing nanoparticles. Such sustainable practices could help reduce the overall cost of production [89].

Biocompatibility and immune response, as well as manufacturing and scalability, represent significant barriers to the clinical translation of nanomaterial-based drug delivery systems. The potential for toxic reactions, immune system activation, and tissue accumulation must be carefully evaluated, while advancements in nanoparticle synthesis, production scalability, and regulatory approval pathways are essential to make these systems viable for widespread clinical use. Overcoming these challenges will require a multidisciplinary approach involving material scientists, engineers, regulatory bodies, and clinicians, working together to ensure the safety, efficacy, and commercial success of nanomaterial-based therapies in autoimmune diseases and beyond [90].

### 4.3. Regulatory Hurdles and Approvals

The clinical translation of nanomaterial-based drug delivery systems faces significant regulatory hurdles. Regulatory agencies such as the U.S. Food and Drug Administration (FDA), European Medicines Agency (EMA), and other national regulatory bodies have established guidelines to ensure the safety, efficacy, and quality of new drug products. However, the approval process for nanomedicines remains complex and challenging due to the unique characteristics of nanomaterials, such as their small size, surface properties, and potential for long-term accumulation in the body [91]. The following section discusses the key regulatory challenges in the approval of nanomaterial-based drug delivery systems and the current state of regulations governing their clinical application.

Challenges in Regulatory Approval of Nanomaterials

➢ Lack of Clear and Unified Guidelines
  One of the primary challenges in the regulatory approval of nanomaterials is the lack of clear, universally accepted guidelines for their evaluation. While regulatory agencies such as the FDA have issued specific guidance on nanotechnology-based products, the regulatory framework is still evolving. For example, the FDA’s “Guidance for Industry: Considering Whether an FDA-Regulated Product Involves the Application of Nanotechnology” (2014) provides recommendations for the assessment of nanomaterial-based drug delivery systems. However, this guidance does not offer a definitive set of regulations or standardized protocols for the safety assessment of nanoparticles across all applications. The lack of uniformity in global regulations complicates the approval process for developers seeking international market access [92].
➢ Safety and Toxicity Testing
  Nanoparticles often exhibit unique biological behaviors compared to their bulk counterparts. Their small size, high surface area, and ability to penetrate biological barriers can result in unintended toxicity or accumulation in tissues, which necessitates thorough safety testing. Regulatory agencies require extensive preclinical and clinical studies to evaluate the biocompatibility, toxicity, and long-term effects of nanoparticles on human health. These studies typically involve in vitro assays, animal models, and clinical trials to assess factors such as cellular uptake, distribution, metabolism, excretion, and any potential adverse effects. However, the safety profiles of some nanoparticles are still not fully understood, and standard toxicity testing methods for nanoparticles are not universally established, leading to delays in regulatory approval [93].
➢ Characterization and Quality Control
  Nanoparticles can exhibit a wide range of properties, including variations in size, surface charge, shape, and stability, which can significantly affect their interactions with biological systems and their therapeutic performance. Regulatory agencies require robust characterization data to ensure the quality and consistency of nanomaterials used in drug delivery. Parameters such as particle size distribution, surface chemistry, drug encapsulation efficiency, and stability must be rigorously assessed. However, the high degree of variability in nanoparticle properties, depending on the synthesis methods and raw materials, makes it difficult to ensure consistency across batches. Inadequate quality control during manufacturing may lead to discrepancies in the safety and efficacy of the final product, potentially delaying regulatory approval [94].
➢ Risk of Nanoparticle Accumulation and Long-Term Effects
  Another challenge in the regulatory approval process is the potential for nanoparticles to accumulate in various organs or tissues over time, leading to toxicity or adverse health outcomes. Nanoparticles, especially those made from non-biodegradable materials, may not fully degrade within the body, posing risks of chronic toxicity or inflammatory responses. Regulatory agencies require long-term studies to assess the biodistribution, clearance, and potential accumulation of nanoparticles in organs such as the liver, spleen, or kidneys. However, such long-term studies can be time-consuming and costly, adding complexity to the regulatory approval process. Furthermore, the potential for nanoparticles to accumulate in the brain or other sensitive tissues is a concern for certain types of drug delivery systems, particularly for treatments involving the central nervous system [95].
➢ Clinical Trial Design and Endpoint Selection
  Clinical trials for nanomaterial-based drug delivery systems are another significant hurdle in the approval process. Designing appropriate clinical trials that can effectively demonstrate the safety and efficacy of nanoparticles is complex. Given the novel nature of nanomedicines, traditional clinical trial designs may not be suitable for evaluating their effectiveness. Nanoparticles may exhibit unique pharmacokinetics and biodistribution profiles that necessitate the development of new biomarkers, imaging techniques, and endpoints to assess their therapeutic effects. Additionally, it can be difficult to predict how nanoparticles will interact with individual patients, as these interactions can vary depending on factors such as genetic makeup, immune system response, and disease state [96].
➢ Regulatory Pathway for Combination Products
  Many nanomaterial-based drug delivery systems are combination products, meaning they involve both a drug and a medical device component (e.g., nanoparticles carrying a therapeutic agent). This dual nature presents challenges in determining the appropriate regulatory pathway. In the U.S., combination products are regulated by the FDA under a separate framework, which may require coordination between different divisions within the agency (e.g., the Center for Drug Evaluation and Research (CDER) and the Center for Devices and Radiological Health (CDRH)). Similarly, in Europe, combination products may fall under the jurisdiction of both the European Medicines Agency (EMA) and the European Commission, creating regulatory complexities. This regulatory overlap can lead to delays in approval and add to the complexity of the clinical development process [97].

Strategies to Overcome Regulatory Challenges

➢ Development of Harmonized Guidelines
  One of the most critical steps in overcoming regulatory hurdles is the development of harmonized, international guidelines for the evaluation of nanomaterials. Regulatory agencies around the world need to establish consistent criteria for the safety testing, characterization, and clinical evaluation of nanomaterial-based drug delivery systems. This would reduce the complexity for developers seeking approval in multiple markets and streamline the regulatory process [98].
➢ Use of Advanced Testing Methods
  The development of advanced testing methods, including high-throughput screening, in silico models, and organ-on-a-chip platforms, can help predict the safety and efficacy of nanomaterials more efficiently. These methods can provide more accurate insights into the behavior of nanoparticles in the body, allowing for better risk assessments and faster approval timelines. In particular, the use of humanized models for testing nanoparticle interactions could reduce the reliance on animal studies and expedite the process [99].
➢ Collaboration between Regulators and Industry
  Increased collaboration between regulatory agencies and industry stakeholders, including nanomaterial developers, pharmaceutical companies, and medical device manufacturers, can help identify and address regulatory challenges early in the development process. By providing clear guidance and fostering communication, regulators can help ensure that nanomaterial-based drug delivery systems meet the necessary safety and efficacy standards while also supporting innovation in nanomedicine [100].
➢ Pre-Clinical and Clinical Study Design Innovation
  Innovations in clinical trial design, such as adaptive trial designs and the use of personalized medicine approaches, could help accelerate the approval process for nanomaterials. Adaptive trials, which allow for modifications based on interim results, could be particularly useful for assessing the efficacy of nanoparticle-based therapies in specific patient populations. Additionally, the integration of personalized medicine, where patients are treated based on their unique genetic and biological profiles, could help optimize the use of nanomaterial-based therapies [101].
  Regulatory hurdles remain one of the most significant barriers to the clinical translation of nanomaterial-based drug delivery systems. The lack of standardized guidelines, safety concerns, and the complexity of clinical trial designs all contribute to delays in approval. However, advancements in testing methodologies, the development of international regulatory frameworks, and increased collaboration between industry and regulatory bodies can help address these challenges and pave the way for the successful commercialization of nanomedicines. As nanotechnology continues to evolve, the regulatory landscape will need to adapt to ensure that nanomaterial-based therapies are safe, effective, and accessible to patients [102].

## 5. Future Perspectives

Nanomaterial-based drug delivery systems have shown immense promise in the treatment of autoimmune diseases, and as technology progresses, their potential to revolutionize healthcare continues to expand. The future of nanomedicines lies in overcoming current limitations and advancing nanomaterial design to meet the ever-evolving needs of autoimmune therapy. Additionally, integrating nanotechnology with personalized medicine approaches offers the opportunity to create more precise, effective, and patient-specific treatments. This section explores the future directions in the field, focusing on the advancements in nanomaterial design and the integration of nanomedicine with personalized approaches to autoimmune disease therapy [59].

### 5.1. Advancements in Nanomaterial Design for Autoimmune Therapy

The design of nanomaterials is at the forefront of innovations in autoimmune disease treatment. The future of nanomedicines for autoimmune diseases will focus on improving the therapeutic efficiency, targeting specificity, and safety profiles of these materials. Several advancements are expected to play a pivotal role in shaping the future landscape of nanomaterial-based drug delivery for autoimmune disorders [103].

#### 5.1.1. Targeted and Site-Specific Drug Delivery

Current efforts in nanomaterial design aim to enhance the targeting of specific immune cells, tissues, or organs involved in autoimmune diseases. Advanced targeting strategies could include the use of ligand-functionalized nanoparticles that bind selectively to receptors expressed on activated immune cells. These targeted approaches are expected to increase the bioavailability of therapeutic agents at disease sites while minimizing off-target effects and systemic toxicity. Moreover, the development of nanomaterials that can simultaneously target multiple molecular pathways could offer an approach for addressing the complex, multi-faceted nature of autoimmune diseases, such as rheumatoid arthritis (RA), lupus, and multiple sclerosis (MS) [104].

#### 5.1.2. Multifunctional Nanomaterials

Another promising avenue for the future of autoimmune therapy is the design of multifunctional nanomaterials capable of performing multiple tasks. For instance, nanoparticles could be engineered to not only deliver drugs but also serve as imaging agents for real-time monitoring of treatment efficacy. Incorporating imaging modalities such as magnetic resonance imaging (MRI) or positron emission tomography (PET) into nanocarriers could provide insights into the biodistribution and accumulation of nanoparticles in the body. Additionally, multifunctional nanomaterials could be designed to release multiple therapeutic agents in response to different stimuli or control the release of drugs over extended periods, further improving the treatment outcomes of autoimmune diseases [105].

#### 5.1.3. Biodegradable and Biocompatible Nanomaterials

Future advancements in nanomaterial design will likely focus on developing biodegradable materials that minimize long-term accumulation in the body. These nanomaterials will be designed to degrade into non-toxic by-products that can be easily eliminated from the body after drug delivery, reducing the potential for chronic toxicity or immune system complications. Advanced polymerization techniques, such as the synthesis of polymers that undergo controlled degradation upon exposure to physiological stimuli (e.g., enzymes or pH changes), are likely to be central to the development of these materials. Additionally, nanomaterials derived from natural or biocompatible sources, such as lipids or polysaccharides, could be increasingly utilized due to their enhanced safety profiles [106].

#### 5.1.4. Smart Nanomaterials with Adaptive Response

The future of nanomaterials for autoimmune therapy will also see the integration of “smart” or “adaptive” systems. These materials will have the ability to respond dynamically to changes in the microenvironment, such as variations in pH, temperature, or enzyme concentration, which are commonly found in inflamed tissues. For example, pH-sensitive nanoparticles could release drugs specifically at the site of inflammation, where the local pH is typically lower than in healthy tissues. Similarly, redox-sensitive nanoparticles could be used to target areas with high oxidative stress, a characteristic of several autoimmune diseases. By incorporating adaptive mechanisms into nanomaterial design, drug release can be tightly controlled and localized, improving the therapeutic effect while minimizing side effects [107].

#### 5.1.5. Nanoparticles with Immune-Modulating Properties

As autoimmune diseases involve dysregulated immune responses, an emerging area of interest is the development of nanoparticles with immune-modulating properties. These nanoparticles could actively interact with the immune system to either enhance or suppress immune responses, depending on the therapeutic needs. For example, nanoparticles could be designed to suppress overactive immune cells responsible for the pathogenesis of diseases like rheumatoid arthritis, or conversely, activate immune responses to combat infection or cancer in autoimmune patients with compromised immunity. These immune-modulating nanoparticles could potentially replace traditional immunosuppressive therapies, which often lead to unwanted side effects such as increased susceptibility to infections [108].

Table 3 presents a comparative overview of the advancements in nanomaterial design for autoimmune therapy, highlighting key nanomaterials and their respective applications in treating autoimmune diseases. Polymeric nanoparticles are particularly valuable for targeted drug delivery, offering high drug loading capacities and the ability to be surface-functionalized for specific targeting, which is useful in diseases like rheumatoid arthritis (RA) and multiple sclerosis (MS). Liposomes, known for their biocompatibility and ability to encapsulate both hydrophilic and hydrophobic drugs, are widely used for the localized delivery of biologics and corticosteroids. However, their stability remains a challenge due to leakage and aggregation issues. Hydrogels are ideal for sustained, localized drug release and mimic biological tissue environments, making them effective in RA treatment; however, their mechanical strength can limit broader applications. Stimuli-responsive systems, such as pH-responsive nanoparticles, enable targeted release in acidic disease environments, a common feature in inflammatory autoimmune diseases, while redox-responsive and enzyme-responsive systems provide even more specificity in disease targeting. Lastly, nanomaterials like carbon nanotubes and dendrimers are showing promise in immune modulation and drug encapsulation, which can improve therapeutic outcomes by addressing immune system dysregulation [109]. Despite the advancements, challenges such as toxicity, stability, and scaling up production remain important considerations for clinical application.

### 5.2. Integration with Personalized Medicine Approaches

Personalized medicine, which tailors medical treatment to individual patients based on their genetic, environmental, and lifestyle factors, is poised to play a significant role in the future of autoimmune disease treatment. Integrating nanotechnology with personalized medicine can offer highly specific and customized treatment strategies that maximize efficacy and minimize adverse effects. Several key areas of integration are likely to drive the future of nanomedicines in autoimmune therapy [111].

#### 5.2.1. Genomic and Proteomic Approaches for Precision Nanomedicine

One of the most promising aspects of personalized medicine is the use of genomic and proteomic data to guide treatment decisions. Nanomaterial-based drug delivery systems could be designed to target specific genetic or proteomic biomarkers associated with autoimmune diseases. For instance, patients with rheumatoid arthritis may have unique patterns of gene expression or protein markers that could be targeted using nanoparticles tailored to recognize these specific molecules. By utilizing patient-specific biomarkers, nanomedicines could be engineered to deliver the most appropriate therapeutic agents based on individual disease profiles, leading to more effective treatments and fewer side effects [112].

#### 5.2.2. Nanomaterial-Based Diagnostic Tools for Personalized Monitoring

Nanomaterials are already being explored for their diagnostic capabilities, such as biosensors for detecting specific biomarkers associated with autoimmune diseases. Integrating diagnostic nanomaterials with therapeutic nanoparticles could enable a real-time, personalized approach to monitoring disease progression and treatment efficacy. For example, nanoparticles could be designed to release therapeutic agents only when specific biomarkers associated with inflammation or autoimmunity are detected in a patient’s bloodstream. This combination of diagnostics and therapeutics, often referred to as “theranostics”, could allow for more precise and dynamic management of autoimmune diseases [113].

#### 5.2.3. Tailored Drug Release Based on Patient Characteristics

Personalized medicine also includes the ability to adjust drug delivery based on individual patient characteristics, including genetic factors, immune responses, and disease severity. Nanomaterials with stimuli-responsive features could be customized to release drugs based on the patient’s specific disease state. For example, in a patient with autoimmune disease flare-ups, a nanomaterial could be engineered to respond to the elevated local inflammatory markers, ensuring that the therapeutic agents are released only when needed. Such tailored drug delivery systems could optimize treatment regimens, enhancing therapeutic outcomes while minimizing adverse effects [114].

#### 5.2.4. Patient-Specific Nanocarriers for Targeted Therapy

Advances in nanotechnology may also facilitate the creation of patient-specific nanocarriers. With the help of 3D printing technologies, personalized nanoparticles could be designed based on a patient’s specific anatomical and physiological features. This approach would allow for precise targeting of the drug delivery system to affected areas, improving therapeutic outcomes and reducing the need for generalized treatments. Personalized nanocarriers could also be developed using biocompatible materials sourced from the patient’s own tissues, further reducing the risk of immune rejection and ensuring higher treatment compatibility [115].

#### 5.2.5. Integration with Digital Health Technologies

The future of personalized nanomedicine may also involve the integration of digital health technologies, such as wearable devices, to continuously monitor patients’ health status and adjust nanomedicine treatment regimens accordingly. Wearables could collect real-time data on inflammation levels, immune system activity, or drug concentrations in the body, which could then be used to adjust the release rates or dosing schedules of nanoparticle-based therapies. This closed-loop system could provide more dynamic and individualized treatment regimens, further improving the precision of autoimmune disease management [116]. The future of nanomaterials in autoimmune disease treatment holds exciting potential, with advances in nanomaterial design paving the way for more efficient, targeted, and personalized therapies. The continued development of multifunctional, biodegradable, and smart nanomaterials, combined with cutting-edge techniques in personalized medicine, promises to revolutionize the management of autoimmune diseases. By enabling precision-targeted therapies and improving patient-specific treatment regimens, nanomedicines could significantly enhance the quality of life for patients suffering from these complex and chronic conditions [117].

### 5.3. Potential for Combining Stimuli-Responsive Nanomaterials with Other Therapeutic Modalities

The combination of stimuli-responsive nanomaterials with other therapeutic modalities offers a promising strategy to enhance the efficacy and specificity of treatments for autoimmune diseases. By integrating multiple therapeutic approaches, it is possible to synergistically target the underlying pathophysiology of autoimmune disorders, overcome the limitations of single treatments, and improve patient outcomes. This section explores the potential for combining stimuli-responsive nanomaterials with other therapeutic modalities, such as traditional drug therapy, gene therapy, immunotherapy, and photothermal or photodynamic therapies, to create more personalized, efficient, and multifaceted treatments for autoimmune diseases.

#### 5.3.1. Combination with Traditional Drug Therapies

Traditional drug therapies, such as non-steroidal anti-inflammatory drugs (NSAIDs), corticosteroids, and immunosuppressants, have been widely used to manage autoimmune diseases. However, these treatments often come with significant side effects, such as immunosuppression, which increases the risk of infections and other complications. Stimuli-responsive nanomaterials offer the potential to improve the delivery and release of traditional drugs, enhancing their therapeutic efficacy while minimizing systemic side effects [118].

##### Enhanced Drug Delivery and Targeting

Stimuli-responsive nanomaterials can be engineered to release their payloads in response to specific physiological signals, such as changes in pH, temperature, or the presence of certain enzymes. For example, nanoparticles designed to be pH-sensitive can release immunosuppressive drugs specifically at inflamed tissues where the pH is lower than in healthy tissues. This approach would reduce the systemic exposure to the drug, minimizing the risk of side effects, while simultaneously increasing drug concentrations at the target site. Combining stimuli-responsive nanomaterials with traditional drugs such as methotrexate, corticosteroids, or biologics could offer a more controlled, site-specific release of therapeutic agents, improving treatment outcomes in autoimmune diseases [119].

##### Co-Delivery of Multiple Therapeutic Agents

A key advantage of stimuli-responsive nanomaterials is their ability to co-deliver multiple therapeutic agents simultaneously. For instance, nanocarriers can be loaded with a combination of immunosuppressive agents and anti-inflammatory drugs, allowing for synergistic effects in controlling immune responses. This approach can be particularly beneficial in autoimmune diseases where both immune suppression and inflammation control are required, such as in rheumatoid arthritis (RA) or systemic lupus erythematosus (SLE). By combining multiple drugs in a single nanoparticle system, it is possible to improve the therapeutic outcomes and reduce the need for polypharmacy, thereby decreasing the likelihood of adverse drug interactions [120].

#### 5.3.2. Integration with Gene Therapy

Gene therapy, which involves the introduction or alteration of genetic material within a patient’s cells to treat disease, is an emerging treatment strategy for autoimmune diseases. However, challenges such as low delivery efficiency, transient gene expression, and potential immune responses limit its widespread use. Stimuli-responsive nanomaterials offer the potential to improve the delivery and effectiveness of gene therapy by providing a targeted, controlled, and non-toxic delivery system.

##### Targeted Delivery of Nucleic Acids

Nanomaterials can be designed to encapsulate nucleic acids such as plasmids, siRNA, or CRISPR/Cas9 constructs, which can then be delivered to specific immune cells or tissues involved in autoimmune diseases. Stimuli-responsive properties allow for the controlled release of nucleic acids at the disease site, ensuring that the genetic material is released in response to environmental cues. For example, in a patient with RA, nanoparticles could be engineered to release siRNA targeting pro-inflammatory cytokines or immune modulators only at the inflamed joint, minimizing systemic gene delivery and reducing the risk of off-target effects [121].

##### Combination with Immune Regulation

Gene therapy using stimuli-responsive nanomaterials could also be combined with immune-regulatory approaches. For example, nanomaterials could deliver genes encoding immune-modulating proteins such as interleukin-10 (IL-10) or transforming growth factor-beta (TGF-β) directly to inflamed tissues, where the presence of local stimuli triggers their release. This approach could help restore immune balance and tolerance, a key aspect of autoimmune disease management. By combining gene therapy with stimuli-responsive nanomaterials, the precision and efficiency of gene delivery can be enhanced, improving therapeutic outcomes while reducing the risk of unwanted immune responses [122].

#### 5.3.3. Synergy with Immunotherapy

Immunotherapy has gained significant attention in the treatment of autoimmune diseases due to its ability to modulate the immune system and promote tolerance. While immune checkpoint inhibitors and monoclonal antibodies have been effective in some autoimmune disorders, their widespread use is limited by challenges such as poor tissue penetration and potential systemic side effects. Stimuli-responsive nanomaterials can enhance the delivery and effectiveness of immunotherapeutic agents by ensuring precise targeting and controlled release.

##### Targeted Immune Modulation

Stimuli-responsive nanomaterials can be designed to deliver immune-modulating agents, such as checkpoint inhibitors, cytokines, or immune-stimulating antibodies, in response to specific microenvironmental cues. For instance, nanoparticles could release immune checkpoint inhibitors only in areas of tissue inflammation or where autoimmune responses are active, thereby enhancing immune tolerance at the site of disease without affecting the rest of the body. This strategy has the potential to reduce the side effects typically associated with systemic immune modulation and increase the effectiveness of immunotherapy in autoimmune diseases like RA, multiple sclerosis (MS), or inflammatory bowel disease (IBD) [123].

##### Co-Delivery with Other Immunomodulatory Agents

Stimuli-responsive nanomaterials can also be used to co-deliver a combination of immunomodulatory agents, such as cytokines or antigen-presenting cell (APC) modulators, to promote immune tolerance. By delivering both immune-activating and immune-suppressing agents simultaneously, nanomaterials can create a balanced immune response that helps to manage autoimmune diseases. This approach may involve using nanoparticles that are responsive to inflammatory cytokines or oxidative stress markers, ensuring that drug release is triggered only in areas where immune dysregulation occurs [60].

#### 5.3.4. Combination with Photothermal and Photodynamic Therapies

Photothermal therapy (PTT) and photodynamic therapy (PDT) are emerging modalities that utilize light to trigger localized treatment effects. Photothermal therapy relies on nanoparticles that convert light energy into heat to selectively kill targeted cells, while photodynamic therapy uses light-activated photosensitizers to generate reactive oxygen species (ROS) to destroy abnormal cells. Both therapies have shown promise in cancer treatment and are now being explored for autoimmune diseases, particularly for their potential to modulate inflammation and immune responses [124].

##### Photothermal Therapy for Inflammation Control

Stimuli-responsive nanomaterials can be combined with photothermal therapy to control inflammation and promote tissue healing in autoimmune diseases. Nanoparticles capable of absorbing light and converting it into heat can be engineered to accumulate at sites of inflammation. Upon exposure to specific wavelengths of light, these particles generate localized heat, which can promote vasodilation, reduce local immune cell infiltration, and induce the death of hyperactive immune cells. This combination of photothermal effects and stimuli-responsive drug delivery could lead to more efficient and targeted therapies for diseases like rheumatoid arthritis or systemic lupus erythematosus [125].

##### Photodynamic Therapy for Immune Modulation

In combination with photodynamic therapy, stimuli-responsive nanomaterials can be used to target and activate immune cells involved in autoimmune diseases. Photosensitizers can be incorporated into nanoparticles that respond to specific stimuli, such as the acidic microenvironment of inflamed tissues. When exposed to light, these photosensitizers generate ROS, which can induce localized immunomodulation by modulating immune cell behavior and promoting the resolution of inflammation. This combination approach holds the potential to not only control the inflammatory response but also to stimulate immune tolerance, offering a novel approach to managing autoimmune diseases. The combination of stimuli-responsive nanomaterials with other therapeutic modalities presents a promising strategy to enhance the treatment of autoimmune diseases. By integrating targeted drug delivery, gene therapy, immunotherapy, and photothermal or photodynamic therapies, it is possible to create more personalized, effective, and multifaceted treatment strategies. These combination therapies have the potential to overcome the limitations of traditional treatments, improve targeting specificity, and minimize systemic side effects. As research continues, the synergistic effects of these combined approaches could revolutionize the way autoimmune diseases are treated, offering new hope for patients with these complex and chronic conditions [126].

Table 4 highlights key nanoparticle-based marketed products that are currently utilized in the management of autoimmune diseases. These include a variety of nanoparticle platforms, such as liposomes, polymer-based nanoparticles, and albumin-bound systems, each designed to enhance drug delivery and therapeutic outcomes. For example, liposomal formulations like Doxil and Ambisome reduce systemic toxicity and improve targeting to inflamed tissues, while polymer-based systems like Copaxone modulate immune responses to alleviate symptoms of diseases like multiple sclerosis. Additionally, monoclonal antibody-based nanoparticles, such as Tocilizumab, target specific cytokines like IL-6 to suppress inflammation in conditions like rheumatoid arthritis. These innovative systems underscore the potential of nanotechnology to address the complexities of autoimmune diseases, offering improved efficacy, reduced side effects, and better patient outcomes.

## 6. Conclusions

### 6.1. Summary of Key Findings

In this review, we have explored the transformative potential of biodegradable and stimuli-responsive nanomaterials for targeted drug delivery in the treatment of autoimmune diseases. Autoimmune disorders present significant therapeutic challenges due to their chronic nature, diverse pathophysiological mechanisms, and the need for precision in treatment to avoid systemic toxicity. Traditional therapies often come with side effects that limit their long-term efficacy and safety. Biodegradable nanomaterials offer a promising solution by providing controlled, localized, and targeted delivery of therapeutic agents, minimizing off-target effects and enhancing treatment outcomes. We discussed various types of biodegradable nanocarriers, including polymeric nanoparticles, liposomes, and hydrogels, which have been designed to degrade safely in the body and release therapeutic agents in response to specific environmental cues such as pH, temperature, redox conditions, and enzymatic activity. These nanomaterials hold the potential to not only improve drug delivery efficiency but also to enhance the specificity and duration of treatment. By integrating these materials with stimuli-responsive properties, it is possible to achieve a more targeted and controlled release of drugs at disease sites, thereby addressing the core challenges in autoimmune disease treatment. Additionally, we highlighted how combining these nanomaterials with other therapeutic strategies, such as traditional drug therapies, gene therapy, immunotherapy, and photothermal therapies, can lead to synergistic effects. These combinations have the potential to improve the overall therapeutic efficacy while reducing the need for complex and often harmful multi-drug regimens. The integration of stimuli-responsive nanomaterials with these therapies could provide a more tailored and effective approach, overcoming the limitations of current treatments. Moreover, we examined the applications of stimuli-responsive nanomaterials in the treatment of specific autoimmune diseases, including rheumatoid arthritis, multiple sclerosis, and inflammatory bowel disease. These case studies demonstrate the growing promise of these advanced materials in the targeted treatment of autoimmune diseases, offering a path forward for improved management of these complex conditions.

### 6.2. Final Thoughts on the Role of Biodegradable and Stimuli-Responsive Nanomaterials in Precision Medicine for Autoimmune Diseases

The role of biodegradable and stimuli-responsive nanomaterials in the field of precision medicine for autoimmune diseases is poised to be a game-changer. Precision medicine relies on the understanding of individual variability in genetics, environment, and lifestyle, tailoring treatments to meet the specific needs of each patient. Biodegradable and stimuli-responsive nanomaterials are well suited to this paradigm, as they offer the ability to design highly specific, adaptable drug delivery systems that can respond dynamically to changes in the patient’s condition or disease progression. As we move forward, the development of these nanomaterials will likely focus on overcoming remaining challenges such as scalability, manufacturing complexity, biocompatibility, and regulatory hurdles. The future of precision medicine in autoimmune disease treatment will depend on the ability to integrate these advanced technologies into clinical practice, ensuring that they are not only effective but also safe and accessible for all patients. Furthermore, the continued exploration of combination therapies, where stimuli-responsive nanomaterials are integrated with other modalities such as gene therapy, immunotherapy, and photothermal therapies, holds great promise for advancing treatment options. By addressing multiple therapeutic targets simultaneously, these combinations may offer a more comprehensive solution to autoimmune disease management. In conclusion, biodegradable and stimuli-responsive nanomaterials represent a cutting-edge approach to the treatment of autoimmune diseases, offering the potential for safer, more efficient, and personalized therapies. Their integration into precision medicine could revolutionize the way we manage these chronic and complex diseases, improving patient outcomes and paving the way for new therapeutic strategies. The continued research and development in this area are critical for realizing the full potential of nanomaterials in transforming autoimmune disease treatment and moving towards more individualized, effective healthcare.

## Figures and Tables

**Table 1:** Comparison of stimuli-responsive mechanisms in nanomaterial drug delivery systems.

Stimuli-Responsive System	Mechanism of Action	Advantages	Challenges	Applications in Autoimmune Disease Treatment	Relative References
pH-Responsive Systems	Triggered by acidic environments typically found in inflamed or diseased tissues.	- High specificity for inflamed or tumor areas - Non-toxic release mechanism	- Limited by variability in pH between different patients and conditions	Used in RA for targeted delivery of anti-inflammatory drugs.	[33,34]
Temperature-Responsive Systems	Release triggered by changes in temperature, often in response to external heating.	- Non-invasive - Can be controlled by localized temperature increases	- May be limited to sites where temperature changes can be reliably induced	Used in MS for localized delivery of therapeutic agents.	[36]
Redox-Responsive Systems	Triggered by redox conditions, particularly in areas with high oxidative stress.	- Targeting inflammatory sites with high oxidative stress - Tailored for specific diseases	- Challenges with controlled release - Variability in redox conditions	Applied in RA for corticosteroid release in inflamed joints.	[38]
Enzyme-Responsive Systems	Release controlled by enzymes overexpressed in disease conditions (e.g., collagenases in RA).	- High specificity - Responsive to enzyme profiles of target tissues	- Limited by enzyme activity and availability in certain tissues	Used in RA and MS for delivery of anti-inflammatory agents.	[39]

**Table 2:** Applications of nanomaterials in autoimmune disease treatment.

Autoimmune Disease	Nanomaterial Type	Therapeutic Target	Key Advantages	Challenges	References
Rheumatoid Arthritis (RA)	Polymeric Nanoparticles, Liposomes	Immunosuppressive drugs, Anti-inflammatory agents	Targeted delivery to inflamed joints, reduced systemic side effects	Risk of polymer toxicity, liposome stability issues	[48,49,50]
Multiple Sclerosis (MS)	Hydrogels, Liposomes, pH-Responsive Systems	Immune modulators, Myelin repair agents	Controlled release, targeting CNS inflammation	Blood–brain barrier crossing, hydrogel degradation rate	[53,54,55]
Lupus (Systemic Lupus Erythematosus)	Carbon Nanotubes, Polymeric Nanoparticles	Cytokines, Autoantibodies	Modulation of immune response, targeting of autoantibodies	Cytotoxicity, immune system disruption	[59,60]
Inflammatory Bowel Disease (IBD)	pH-Responsive Nanoparticles, Liposomes	Anti-inflammatory drugs, TNF inhibitors	Targeted drug release at intestinal sites, reduced gastrointestinal side effects	Variability in pH conditions, formulation complexity	[17,61,62]
Psoriasis	Dendrimers, Polymeric Nanoparticles	Anticytokine agents, Immunosuppressive drugs	Localized delivery to skin, enhanced bioavailability	Skin irritation, systemic absorption issues	[58]
Type 1 Diabetes	Hydrogels, Liposomes	Insulin, Immunomodulatory agents	Enhanced glucose control, reduced autoimmunity	Risk of immunogenicity, hydrogel degradation	[61,63,64]

**Table 3:** Advancements in nanomaterial design for autoimmune therapy.

Advancement	Nanomaterial Type	Key Features	Applications in Autoimmune Disease Therapy	Challenges	References
Targeted Drug Delivery	Polymeric Nanoparticles	High drug loading capacity, controlled release, surface functionalization	Used for RA and multiple sclerosis (MS), delivering immunosuppressive agents locally	Risk of polymer toxicity, variability in degradation rates	[103]
Biodegradable and Biocompatible Carriers	Liposomes	Lipid bilayer vesicles, biocompatible, can encapsulate hydrophobic and hydrophilic drugs	Applied in MS for targeted delivery of biologics and corticosteroids	Stability concerns, leakage or aggregation of liposomes	[105]
Hydrogels for Sustained Release	Hydrogels	Water-absorbing networks, mimics biological tissues, provides localized drug release	Used in RA for localized treatment of inflammation	Mechanical strength limitations, slow degradation in certain conditions	[110]
Stimuli-Responsive Drug Release	pH-Responsive Nanoparticles	Release triggered by acidic environments, enhancing specificity	Targeted delivery in inflammatory sites like RA or Crohn’s disease	Variability in pH conditions between patients and disease sites	[71,72,73]
Nano-based Immunomodulation	Carbon Nanotubes, Graphene Oxide	Ability to modulate immune responses, excellent surface area for functionalization	Treatment of autoimmune diseases like lupus or RA, modulating immune cell activity	Potential cytotoxicity, challenges with large-scale synthesis	[107]
Nanoscale Drug Encapsulation	Dendrimers, Micelles	Nanoscale carriers with high surface area, precise drug encapsulation and release	Delivery of corticosteroids or TNF inhibitors in RA	Complexity in synthesis, difficulty in scaling up production	[109]

**Table 4:** Nanoparticle-based marketed products for autoimmune diseases.

Product Name	Nanoparticle Type	Therapeutic Agent	Indication	Manufacturer	Mechanism of Action
Abraxane	Albumin-bound NPs	Paclitaxel	Rheumatoid Arthritis (off-label)	Celgene Corporation	Targeted drug delivery to inflamed tissues
Doxil	Liposomal NPs	Doxorubicin	Systemic Lupus Erythematosus (off-label)	Johnson & Johnson	Reduces inflammation and immune response
Copaxone	Polymer-based NPs	Glatiramer Acetate	Multiple Sclerosis	Teva Pharmaceuticals	Modulates immune system activity
Liposomal Amphotericin B (Ambisome)	Liposomes	Amphotericin B	Autoimmune fungal infections	Gilead Sciences	Reduces toxicity and enhances targeting
Tocilizumab (Actemra)	Liposomal NPs	Monoclonal Antibody	Rheumatoid Arthritis	Roche	Targets IL-6 to reduce inflammation

## Data Availability

No new data were created or analyzed in this study. Data sharing is not applicable to this article.
